# Massive immune response against IVIg interferes with response against other antigens in mice: A new mode of action?

**DOI:** 10.1371/journal.pone.0186046

**Published:** 2017-10-12

**Authors:** Laetitia Sordé, Sebastian Spindeldreher, Ed Palmer, Anette Karle

**Affiliations:** 1 Novartis Pharma AG, Integrated Biologics Profiling Unit, Immunogenicity Risk Assessment, Basel, Switzerland; 2 Novartis Institute for Biomedical Research, Drug Metabolism and Pharmacokinetics, Biologics, Basel, Switzerland; 3 University Hospital Basel, Department of Biomedicine, Transplantation Immunology and Nephrology, Basel, Switzerland; Institut National de la Santeet de la Recherche Medicale (INSERM), FRANCE

## Abstract

Administration of high dose intravenous immunoglobulin (IVIg) is widely used in the clinic to treat autoimmune and severe inflammatory diseases. However, its mechanisms of action remain poorly understood. We assessed the impact of IVIg on immune cell populations using an *in vivo* ovalbumin (Ova)-immunization mouse model. High dose IVIg significantly reduced the Ova-specific antibody response. Intriguingly, the results obtained indicate an immediate and massive immune reaction against IVIg, as shown by the activation and expansion of B cells and CD4+ T cells in the spleen and draining lymph nodes and the production of IVIg-specific antibodies. We propose that IVIg competes at the T-cell level with the response against Ova to explain the immunomodulatory properties of IVIg. Two monoclonal antibodies did not succeeded in reproducing the effects of IVIg. This suggests that in addition to the mouse response against human constant domains, the enormous sequence diversity of IVIg may significantly contribute to this massive immune response against IVIg. While correlation of these findings to IVIg-treated patients remains to be explored, our data demonstrate for the first time that IVIg re-directs the immune response towards IVIg and away from a specific antigen response.

## Introduction

Intravenous immunoglobulin (IVIg) is a natural blood product constituted of a pool of polyclonal IgG purified from several thousand healthy donors. Beyond its initial indication as a replacement therapy for immunodeficient patients, today, treatment of autoimmune and inflammatory diseases account for the vast majority of IVIg administrations [1]. IVIg is mostly used in “off-label” prescriptions in a wide range of autoimmune diseases in fields of rheumatology, neurology, dermatology or hematology [2, 3]. Nevertheless, high dose IVIg therapy is required to achieve immunosuppressive properties (1 to 4 g/kg).

Despite its widespread use, the mechanisms that confer IVIg its immunoregulatory properties in autoimmune conditions remain unclear. Research has focused on identifying anti-inflammatory components of IVIg, and various mechanisms have been suggested. Fab-dependent activities include the presence of natural autoantibodies that recognize and block a wide array of pro-inflammatory molecules, including cytokines [4, 5], leukocyte adhesion molecules [6], Fc-gamma receptors (FcγRs) [7], or complement components [8–10]. Other studies have shown that disease-specific anti-idiotypic antibodies found in IVIg preparations could neutralize pathogenic antoantibodies [11–13], while others indicate that IVIg would be effective through Fc-dependent mechanisms. While administration of high IgG doses have been postulated to saturate the FcRn receptors and thereby accelerate the degradation of circulating pathogenic autoantibodies [14–16], a recent study in a murine model of ITP (immune thrombocytopenia) reported that IVIg was still effective in increasing platelet counts in the absence of FcRn [17]. It has also been suggested that the immunosuppresive effects of IVIg result from FcγRs blockade after binding of IgG antibodies through their Fc-fragment [18, 19]. A small portion of IgG dimers present in IVIg solutions have been proposed to account for this effect, as they show enhanced avidity for low affinity FcγRs [20, 21]. In contrast, other studies have shown that a human IgG preparation lacking IgG dimers retained its therapeutic efficacy in a murine model of ITP [22], and that IVIg activity was not impaired in FcγRI and FcγRIII-deficient mice [23]. Among the proposed hypotheses, some studies demonstrated that IVIg up-regulated the inhibitory FcRγIIB on splenic macrophages in mice [24, 25] or on monocytes and B cells in human [26]; furthermore other studies showed that FcRγIIB deficiency abrogated anti-inflammatory effects of IVIg [27–29]. Still, the relevance of FcRγIIB in IVIg therapeutic efficacy has been questioned in several murine studies where FcRγIIB was dispensable [30–32], as well as in patients where IVIg treatment did not modify FcRγIIB expression in monocytes [33, 34]. Alternatively, several groups have suggested that 2 to 4% of IgG antibodies with Fc domains carrying α2,6-linked sialylated *N*-glycans represent the anti-inflammatory fraction of IVIg [35–39]. However, the paradigm of sialylation exerting the immunosuppressive effects of IVIg remains under debate as other reports have demonstrated that sialylation was dispensable for IVIg-mediated inflammation resolution [21, 40–44].

In the light of the multitude of proposed mechanisms and the controversy in the field, the mechanism by which IVIg mediates its immunosuppressive activity remains an open question. This work aimed to study the direct impact of IVIg on the different immune cell populations using a mouse ovalbumin (Ova) sensitization model.

IVIg is used to treat autoimmune diseases which would be reflected by a mouse model with an already established inflammatory response such as AIA (Antigen-induced arthritis), EAE (Experimental autoimmune encephalomyelitis) or CIA (Collagen-induced arthritis). On the other hand, IVIg is also used in the scope of replacement therapies to reduce immunogenicity against the biotherapeutic administered to the patient [45–47]. The mechanism of such a co-medication is adequately reflected in sensitization models and therefore suggests that a sensitization model could be used to investigate if and how IVIg given in addition to a protein could inhibit unwanted immunogenicity against this protein. Therefore, an ova-sensitization model was used to investigate the effects of IVIg in the scope of this study.

While IVIg efficiently inhibited the production of anti-Ova antibodies, we demonstrate that IVIg triggered a massive immune activation and recruitment of immune cells that could not be reproduced with two human monoclonal IgGs. Our results suggest that the enormous sequence diversity present in IVIg introduce a multitude of new foreign epitopes that re-direct the immune system towards a massive immune response against IVIg, consequently diminishing the ability to efficiently respond against other antigens.

## Methods

### Mouse experiments

All animal experiments were performed in accordance with the Federal and Cantonal laws of Switzerland and approved by the Cantonal Veterinary Office of Basel-Stadt. Female mice C57Bl/6NCrl were obtained from Charles River, France. All mice were used at an age of 7–11 weeks.

Mice were immunized 3 times once a week sub-cutaneously in the neck with 50 μg Ova (Invivogen) together with adjuvant AddaVax^™^ (MF59, Invivogen) at 1:5 v/v. NP-OVAL and NP-AECM-Ficoll (Biosearch Technologies) were injected at 50 μg/ mouse. Eventually the following compounds were co-injected as indicated: IVIg (Octagam 10%; Octapharma), bevacizumab (Roche) or panitumumab (Amgen). All injections volumes were completed to 600 μL with DPBS (Gibco Invitrogen). Mice were terminated 24h after the last injection and blood and organs were collected.

### Flow cytometry

Fc-gamma receptors were blocked with a rat anti-mouse CD16/CD32 antibody (BD). Mouse-specific antibodies were the following: CD3-Brilliant violet 510, CD25-PercP Cy5.5, I-A/I-E-Brilliant violet 510, CD45R/B220-PE-Cy7, CD43-APC, CTLA-4-APC (all from BioLegend), CD4-APC-Cy7, CD69-PE, CD11c-PercP Cy5.5, CD19-APC-Cy7, CD40-FITC (all from BD Pharmingen), CD8α-PE-Cy7, FoxP3-FITC, CD86-APC, CD83-FITC, CD80-PE-Cy7, IgM-PercP-eFluor 710 (all from eBioscience).

During the experimental set-up, CD3 antibody was included in the staining panel (BV510 BioLegend clone 17A2) and used for the gating of T cells. The results obtained with gating on CD3+ CD4+ CD8- were similar to those obtained with gating on CD4+ CD8-. Due to the limitation in the maximum number of colours that can be discriminated with the FACS Canto II, CD3 staining was omitted in subsequent stainings.

Dead cells were excluded using the fixable viability dye-eFluor 450 (eBioscience), and intracellular staining was performed using the fixation/ permeabilization buffer set from eBioscience. Flow cytometry measurements were performed on a FACS Canto II instrument (BD) and the data were analyzed with FlowJo software (Tree Star).

### ELISA

Mouse Ova-specific IgG were measured following the instructions provided by the manufacturer’s kit (Chondrex).

ELISA for measuring anti-IVIg IgG were conducted using either IVIg (Octagam 10%; Octapharma), IVIg-Fc or (Fab’_2_) as capture antigens. IVIg-Fc fragments were generated by papain digestion (Roche) and IVIg-(Fab’_2_) fragments by FabRICATOR^®^ digestion (Genovis). A mouse polyclonal IgG anti-human IgG (H+L) (Jackson Immunoresearch) was used as standard. ELISA for total mouse IgG were conducted using a polyclonal goat anti-mouse IgG as capture antibody which had been adsorbed against human sera (not cross-reactive to human IgG), and a mouse IgG as standard (SouthernBiotech). ELISA for anti-NP antibodies was performed using NP-BSA (Biosearch Technologies) as capture antigen. For all assays a polyclonal goat coupled to alkaline-phosphatase anti-mouse IgG or IgM (SouthernBiotech) was used for detection, and the plates were developed with the p-nitrophenyl phosphate substrate (Sigma). OD was acquired at 405 nm on a Versa max reader (Molecular Devices).

Results were analyzed on the Softmax^®^ Pro software (Molecular Devices) and data expressed as relative units compared to the standard.

### Staining of germinal centers by immunofluorescence

After necropsy, organs were frozen in OCT compound (Tissue-Tek^®^; Sakura) and transferred in -80°C. Sections of 5 μm were fixed in PFA 4% (Sigma), and washed in PBS-Tween 0.05%. After blocking with Image-IT FX Signal Enhancer (Life Technologies), GL7-FITC antibody (BD) and PNA-biotin (Sigma) were applied at 10 μg/mL overnight at 4°C. Mouse anti-FITC-Alexa Fluor^®^ 488 antibody (Jackson Immunoresearch) and/ or streptavidin-Alexa Fluor^®^ 647 (Life Technologies) at 1:200 dilution were used for detection. After counterstaining with 2 ug/ml DAPI (Molecular Probes), slides were washed and mounted in Fluoromount^™^ Aqueous Mounting Medium (Sigma) with coverslips. Images were acquired with NanoZoomer 2.0 HT (Hamamatsu) and analyzed with NDP.view2 software (Hamamatsu).

### ELISPOT

96-well nitrocellulose membrane plates (Millipore) were coated with 10 μg/ml polyclonal goat anti-mouse IgG or IgM (SoutherBiotech), or with 5 μg/mL capture antigen (Ova, Invivogen). After washing and blocking with 1% BSA in DPBS, the cellular suspensions obtained from lymphoid organs were applied on the plate at 200 000, 40 000, 8000, or 1600 cells/well. To measure the specific anti-OVA IgG secreting cells, cells were plated in half a plate at 200 000 cells/well. Plates were incubated overnight at 37°C. The plates were washed and incubated with a polyclonal goat biotinylated anti-mouse IgG or IgM (SouthernBiotech), followed by streptavidin-HRP (SouthernBiotech). Plates were developed with AEC substrate (Sigma) and read on a CTL-ImmunoSpot^®^ Analyzer (Cellular Technology Ltd) with the ImmunoCapture^™^ Version 6.1. Software and spots were counted with ImmunoSpot^®^ Professional Version 4.0.

### Statistics

Graphs and statistical analyses were generated with GraphPad Prism 7.0. One- or two-way ANOVA were performed where indicated.

## Results

### IVIg inhibits anti-Ova IgG response *in vivo* but increases numbers of B and T-cells in secondary lymphoid organs

**Fig 1:**
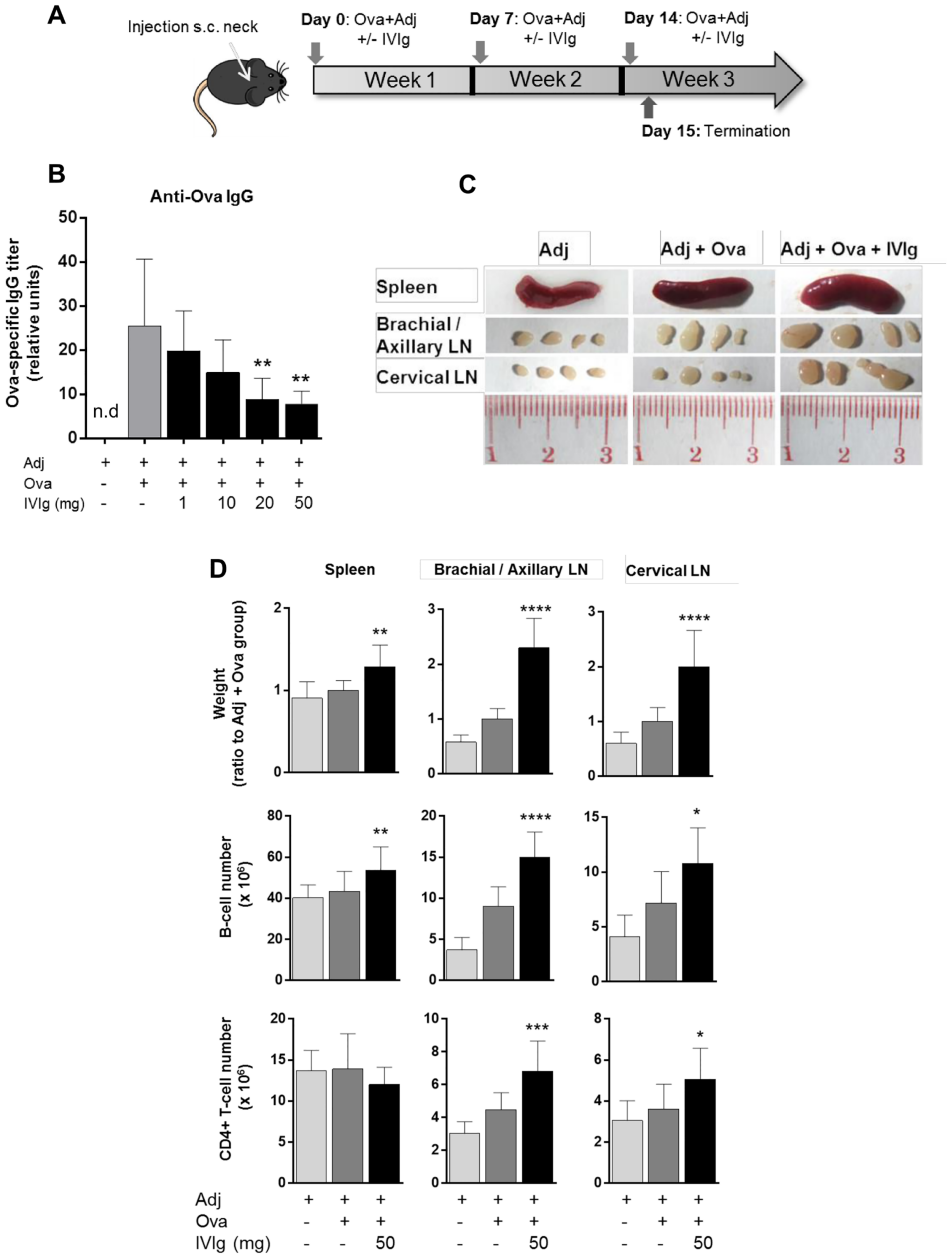
IVIg inhibits anti-OVA IgG response *in vivo* in a dose-dependent manner but increases the weight of spleen and draining lymph nodes. (A) Mouse immunization protocol. C57Bl/6NCrl mice were injected 3 times weekly subcutaneously in the neck with 50 μg Ovalbumin (Ova) and adjuvant AddaVax^®^ (MF59), and terminated 24h after last injection. IVIg doses ranging from 1 mg to 50 mg were co-injected as indicated. (B) IgG mouse response against Ova measured by ELISA performed on mouse serum. Values are expressed as relative units to standard. Results pool data from 2 independent experiments (n = 6). Bars represent mean ± SD. n.d: not detected. Statistical significance was tested using one-way ANOVA (Dunnett’s test) where each group was compared to the control group ‘Adj + Ova’. (C) Pictures of spleen and draining lymph nodes (LN) from one representative animal in each group of treatment. (D) Weight of spleen and draining LN with number of B cells and CD4+ T cells as measured by flow cytometry. For weight, values are expressed as ratio to the ‘Adj + Ova’ treated group. For cell numbers, values are expressed as absolute cell numbers. Dead cells were excluded using a viability fluorescent dye. B cells were gated on CD19+ cells and T cells on CD4+ CD8- cells. Bars represent mean ± SD. Data pool 4 independent experiments (n = 12). Statistical significance was tested using one-way ANOVA (Dunnett’s test) where each group was compared to the control group ‘Adj + Ova’. Star maker significant difference *: p<0.05; **p<0 0.01; ***: p<0.001; ****: p<0.0001. n.d: not detected.

Mice were injected subcutaneously with Ova together with adjuvant AddaVax^®^ (MF59-based). Different doses of IVIg ranging from 1 mg to 50 mg were administered simultaneously at the same injection site (Fig 1A). IVIg administration reduced the amount of secreted Ova-specific mouse IgG, proportionally to the dose injected (Fig 1B). Doses of 50 mg and 20 mg IVIg per animal (equivalent to 2.5 g/kg and 1 g/kg for a mouse of 20 g) resulted in significant inhibition by 69% and 65%, respectively. The inhibitory effect was not significant with doses of 10 mg and 1 mg per animal (equivalent to 500 mg/kg and 50 mg/kg, respectively).

Unexpectedly, treatment with 50 mg of IVIg increased the size of the draining lymph nodes compared with mice that received only Ova + adjuvant (Fig 1C). Spleen weight was slightly but significantly increased upon IVIg treatment, whereas the draining lymph nodes were enlarged by approximately 2-fold (Fig 1D). This increase in organ weight upon IVIg administration was accompanied by an increase in the number of B-cells in both the spleen and draining lymph nodes. Moreover, IVIg treatment resulted in a higher number of CD4+ T-cells in the draining lymph nodes, as compared to the Ova + adjuvant -treated group (Fig 1D). Taken together these data suggest an activation of the immune system by IVIg *in vivo*, as indicated by enlarged lymphoid organs and the increased number of CD4+ T cells and B cells.

### IVIg activates B and T cells in a dose-dependent manner only when co-injected with adjuvant

**Fig 2:**
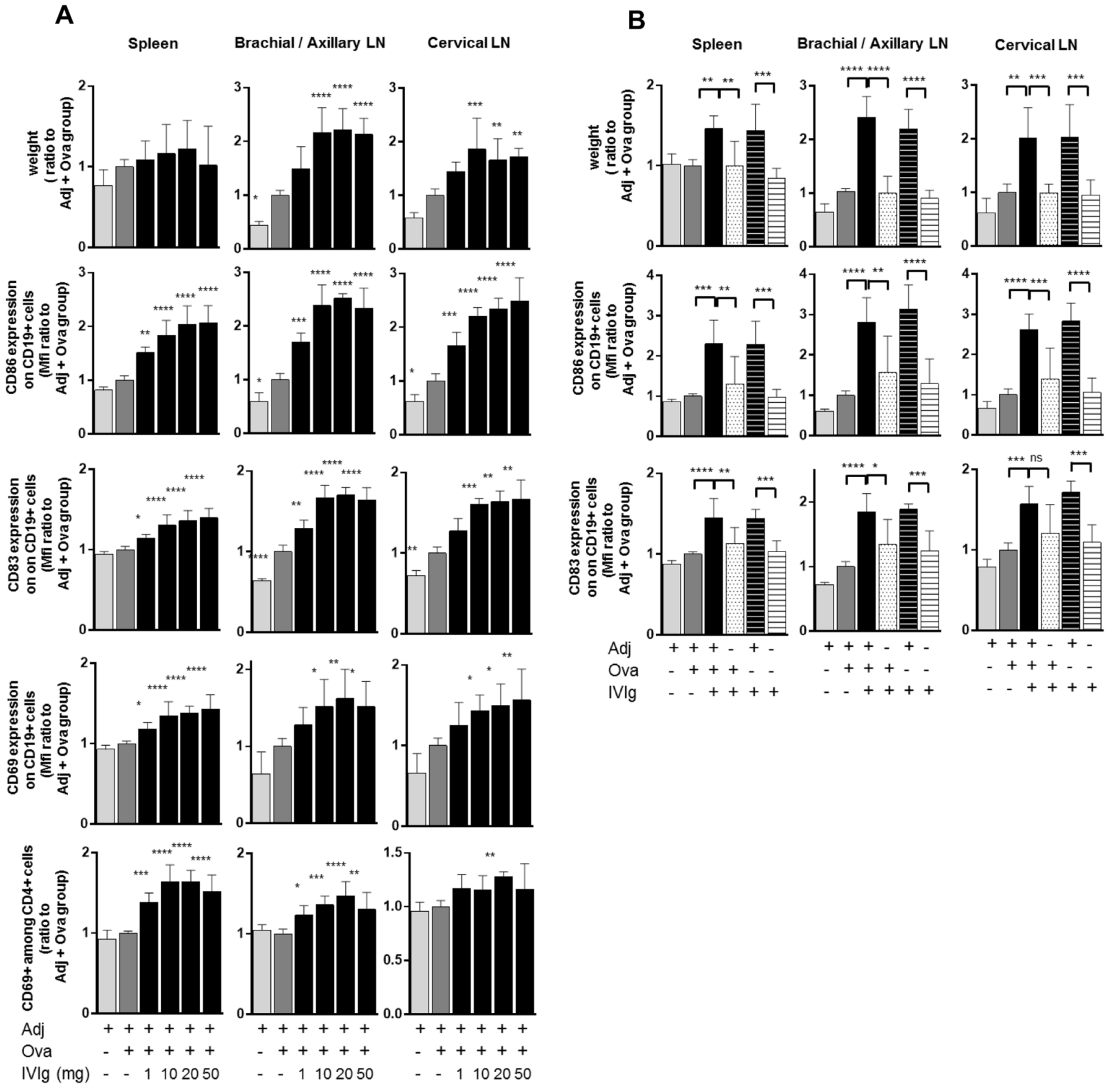
IVIg activates B and T cells in a dose-dependent manner and only when co-injected with adjuvant. (A) Mice were immunized with Ova as described previously and IVIg doses ranging from 1 mg to 50 mg were co-injected. Expression of activation markers and co-stimulatory molecules on B and T cells was measured by flow cytometry. Results pool data from 2 independent experiments (n = 6). Values are expressed as ratio to the ‘Adj + Ova’ treated group. Bars represent mean ± SD. Statistical significance was tested using one-way ANOVA (Dunnett’s test) where each group was compared to the control group ‘Adj + Ova’. (B) Presence of adjuvant is required together with IVIg to mediate increase of weight in lymphoid organs and B cells activation. Mice were immunized with Ova and 50 mg of IVIg was injected alone or in combination to Ova and/or adjuvant AddaVax^®^ (MF59), as indicated. Results are pooled data from 2 independent experiments (n = 6). Values are expressed as ratio to the ‘Adj + Ova’ treated group. Bars represent mean ± SD. Statistical significance was tested using one-way ANOVA (Tukey’s test) where each group was compared with every other. Star maker significance *: p< 0.05; **p<0.01; ***: p<0.001; ****: p<0.0001. MFI: Median of fluorescence intensity. LN: lymph nodes.

To further characterize the effect of IVIg on B and T cells, expression of activation markers was investigated in Ova-immunized mice treated with or without IVIg. Intriguingly, IVIg significantly increased the expression of the co-stimulatory molecules CD86 and CD83 and the early activation marker CD69 on B cells in a dose-dependent manner, in both the spleen and the draining lymph nodes (Fig 2A). CD80 expression on B cells remained unchanged after IVIg treatment and changes in CD40 and MHC class II expression on B-cells were negligible (S1 Fig). IVIg did not affect CD86, CD83 and I-A expression on splenic dendritic cells (DCs) (S2A Fig). IVIg injection in Ova-immunized mice led to a significantly higher proportion of CD4+ CD69+ activated T cells in the spleen and lymph nodes (Fig 2A). The percentage of Tregs in lymphoid organs remained unchanged in IVIg-treated mice, as well as the expression of FoxP3, CD25 and CTLA-4 on CD4+ FoxP3+ Tregs (S2B Fig). This indicates that IVIg had no effect on the phenotype of Tregs, although their functionality was not investigated here. Control experiments showed that the effect of IVIg could not be reproduced with the corresponding formulation buffer control. Moreover, immunization with an alternative antigen, BSA, demonstrated that the effect of IVIG is antigen independent (S3 Fig). In summary, these results indicate that IVIg activated both B cells and effector CD4+ T cells in a dose-dependent manner when co-injected with Ova and adjuvant.

To determine the respective contribution of Ova and adjuvant to these effects mediated by IVIg, mice were treated with different combinations of IVIg, Ova and adjuvant as indicated in Fig 2B. In mice receiving IVIg and adjuvant with or without Ova, the weight of the spleen and draining lymph nodes was elevated, and in both groups expression of CD86 and CD83 on B cells was increased by 2 to 3-fold. These effects could not be reproduced in groups treated with IVIg alone, adjuvant alone, or IVIg and Ova without adjuvant, demonstrating that the presence of adjuvant was required for IVIg to mediate B-cell activation and to increase the weight of lymphoid organs. These effects could be reproduced with five different adjuvants, irrespectively of the type of adjuvant (S4 Fig).

### Human monoclonal antibodies cannot mimic IVIg effects

**Fig 3:**
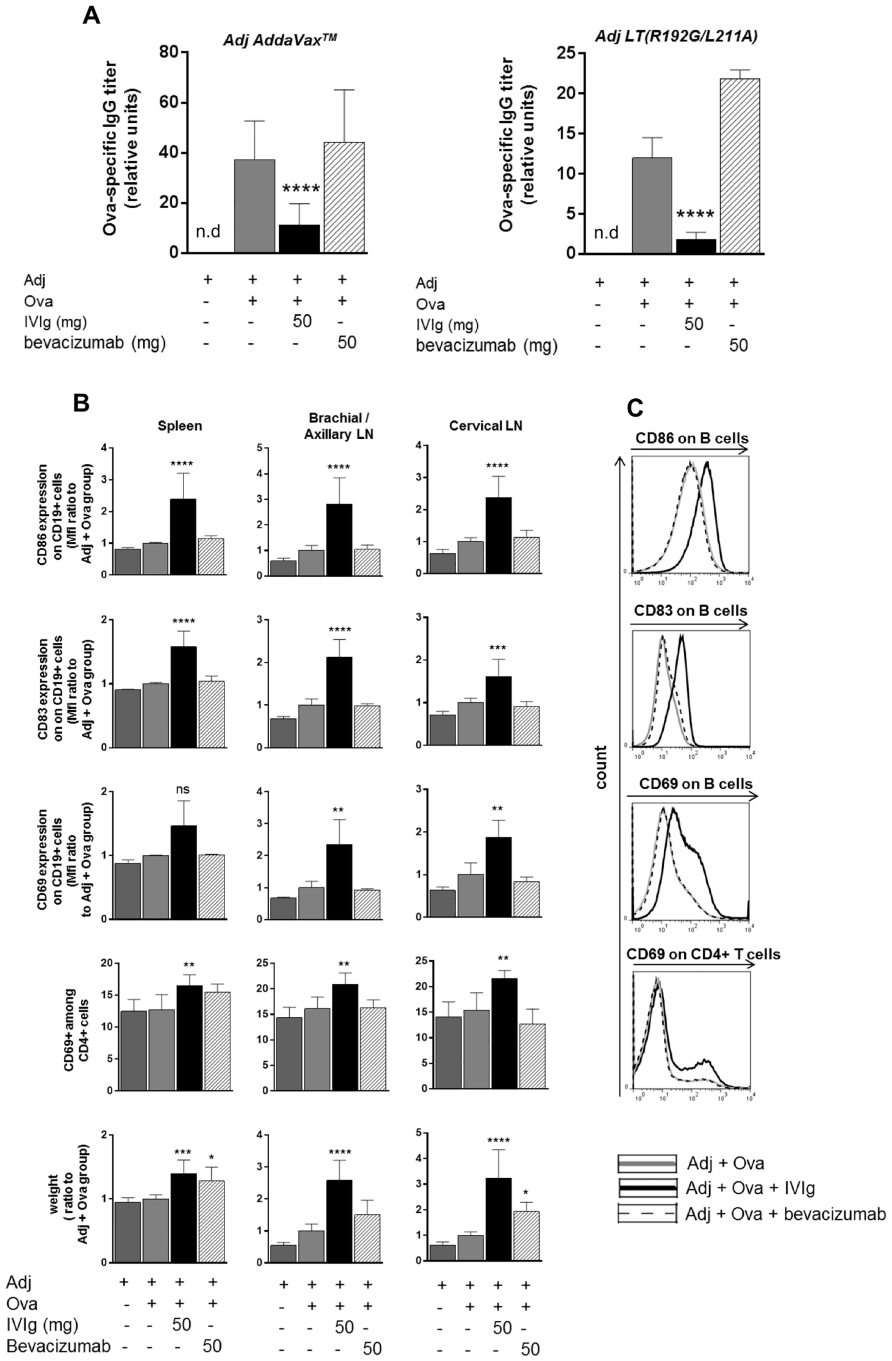
Bevacizumab, a humanized monoclonal IgG_1_κ antibody, does not reproduce IVIg effects. Mice were immunized with Ova as described previously using either adjuvant AddaVax^®^ (MF59) or adjuvant LT(R192G/L211A). IVIg or bevacizumab were co-injected as indicated. (A) Anti-Ova IgGs in mouse serum were measured by ELISA. Values are expressed as relative units to standard. For experiment using adjuvant AddaVax^®^ (MF59), results pool data from 4 independent experiments (n = 12) and for adjuvant LT(R192G/L211A) results are from one experiment (n = 3). Bars represent mean ± SD. Statistical significance was tested using one-way ANOVA (Dunnett’s test) where each group was compared to the control group ‘Adj + Ova’. (B) Draining lymph nodes and spleen were harvested, weighed and flow cytometry was performed on isolated cells (adjuvant AddaVax^®^ was used). Results pool data from 2 independent experiments (n = 6), except for CD69 measured on B cells for which data are derived from one experiment (n = 3). Bars represent mean ± SD. Statistical significance was tested using one-way ANOVA (Dunnett’s test) where each group was compared to the control group ‘Adj + Ova’. (C) Flow cytometry histograms are shown for one representative animal. Star maker significant difference *: p< 0.05; **p<0.01; ***: p<0.001; ****: p<0.0001. ns: not significant. n.d: not detected. MFI: Median of fluorescence intensity. LN: lymph nodes.

To address whether the effects of IVIg could be reproduced by a monoclonal antibody, mice were co-injected with Ova, adjuvant and a marketed humanized IgG_1κ_ antibody, bevacizumab. The absence of cross-reactivity of bevacizumab with murine antigens [48] excludes potential interference with the immune system that could impair the interpretation of the data. While IVIg consistently led to a significant decrease in the anti-Ova-IgG titer, administration of bevacizumab at the same dose had no inhibitory effect on the secretion of Ova-specific antibodies as tested with two different adjuvants (Fig 3A). Unlike IVIg, injection of bevacizumab did not increase the expression of CD86, CD83 and CD69 on B cells nor the expression of CD69 on CD4+ T cells (Fig 3B and 3C). Injection of bevacizumab significantly increased the weight of the spleen and cervical lymph nodes (Fig 3B), which shows some level of mouse immune response against the human protein, as expected. Injection of panitumumab, a marketed human IgG_2κ_, could also not reproduce these effects of polyclonal IVIg (S5 Fig).

Taken together, these data indicate that a monoclonal IgG antibody injected at an equivalently high dose can impair neither an antigen-specific humoral response nor activate the immune system in the same manner as IVIg.

### IVIg induces the formation of numerous germinal centers

**Fig 4:**
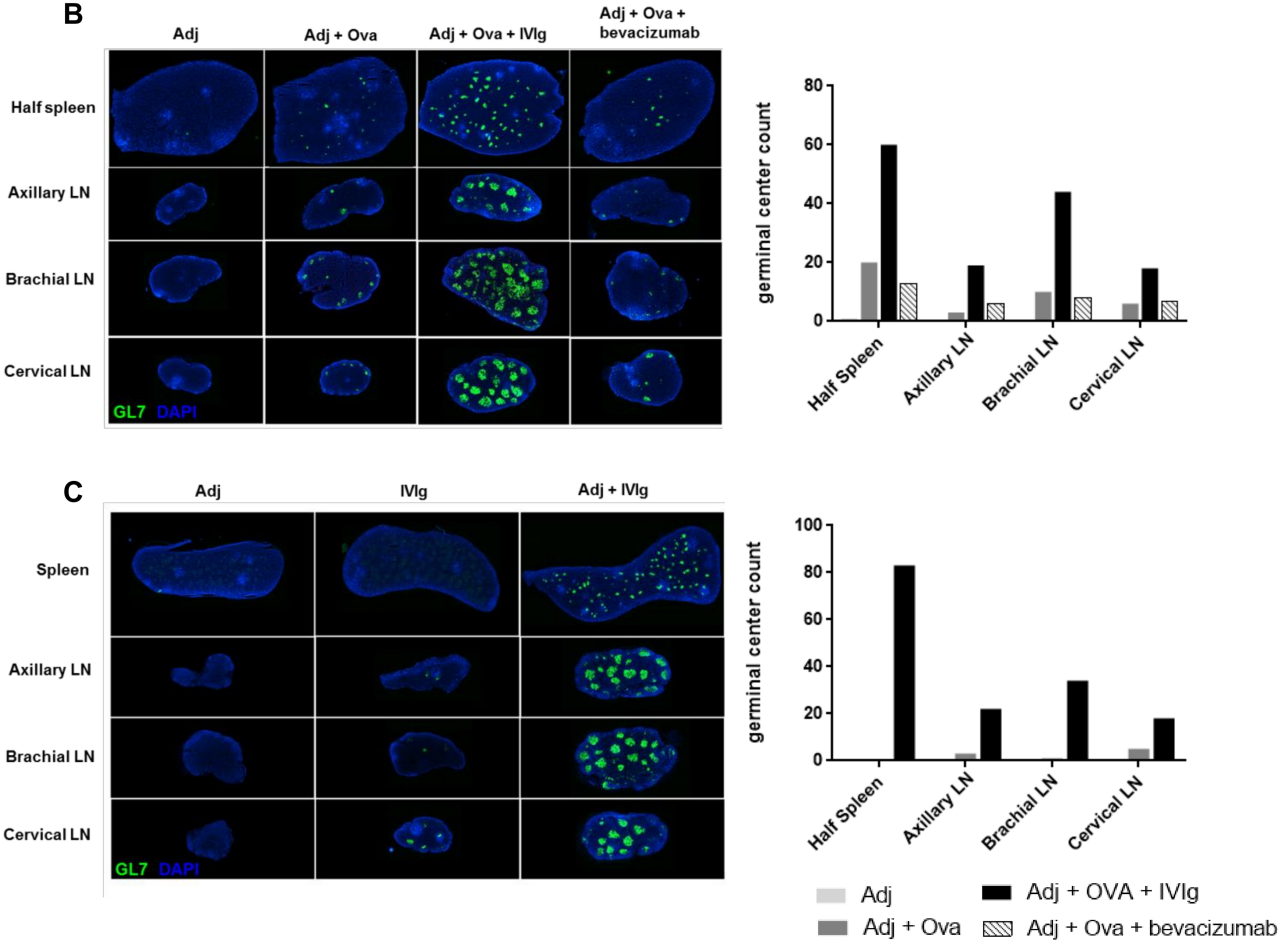
IVIg induces the formation of numerous germinal centers. (A) Spleen sections of Ova-immunized mice were co-stained with PNA (Peanut agglutinin from *Arachis hypogaea*, in red) and the rat monoclonal GL7 antibody (green) to verify specificity of the staining. Magnification: x 20. (B-C) Mice were immunized with Ova as described previously and 50 mg of IVIg or bevacizumab were co-injected as indicated (B) or IVIg was injected alone or in combination to adjuvant AddaVax^®^ (C). Spleen and draining lymph nodes were stained for germinal centers with GL7 antibody (green). Graphs show the number of germinal centers in each group of treatment. Each picture is generated from one representative animal. n = 6 animals from 2 independent experiments for (B) and n = 3 animals for (C). Magnification: x 0.25.

In a next step the formation of germinal centers was evaluated by staining activated B and T cells with the rat monoclonal GL7 antibody in splenic and lymph nodes sections. In a first step (Fig 4A), the specificity of the GL7 antibody was verified by co-staining with PNA (Peanut agglutinin from *Arachis hypogaea*). PNA binds to glycan moieties with a terminal β-galactose residue at the core-1 branch of O-linked glycans, and is commonly used as a marker for germinal center B cells [49]. As expected, injection of adjuvant alone produced no germinal centers, whereas addition of Ova triggered the formation of few and small germinal centers (Fig 4B and S6A Fig). Strikingly, mice that had received IVIg together with Ova and adjuvant exhibited a high number of germinal centers in their spleen and draining lymph nodes along with markedly enlarged organ sizes, the lymph nodes in particular. In line with our previous data, the formation of germinal centers remained unchanged after administration of bevacizumab compared to mice immunized with Ova and adjuvant. Interestingly, the number of germinal centers was strongly augmented in mice receiveing IVIg together with adjuvant, but not when mice received IVIg or adjuvant alone (Fig 4C and S6B Fig). These results confirm that a strong immune response is initiated by IVIg *in vivo* that is dependent on the co-injection of an adjuvant.

### IVIg raises a specific antibody response in mice

**Fig 5:**
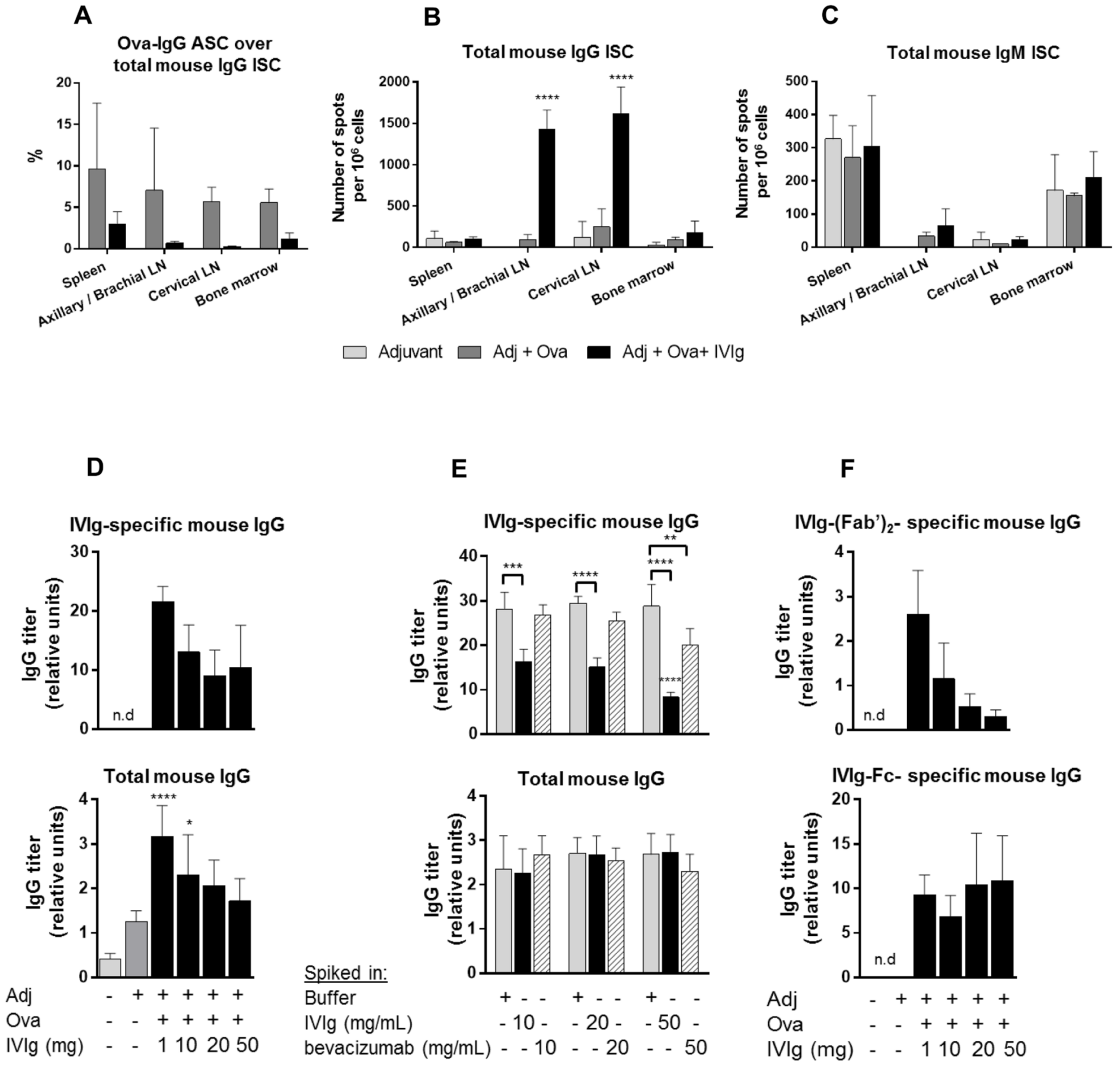
IVIg increases IgG-Immunoglobulin secreting cells in lymph nodes and raises a specific antibody response in mice. (A-C) Mice were immunized with Ova and 50 mg of IVIg were co-injected. Cells were processed from lymphoid organs and plated for ELISPOT assay to assess (A) Proportion of Ova-specific IgG antibody secreting cells (ASC) relative to total IgG immunoglobulin-secreting cells (ISC) (B) Total IgG ISC or (C) Total IgM ISC. Data are derived from one experiment (n = 3). Statistical significance was tested using two-way ANOVA (Sidak’s test for (A) and Dunnett’s test for (B-C) where each group was compared to the control group ‘Adj + Ova’). (D) Mice were immunized with Ova and increasing doses of IVIg were co-injected as indicated. IVIg-specific mouse IgG or total mouse IgG were measured by ELISA. Values as expressed as relative units to standard. Data were pooled from 2 independent experiments (n = 6). Statistical significance was tested using one-way ANOVA (Dunnett’s test) where each group was compared to the control group ‘Adj + Ova’. (E) Addition of IVIg in vitro in mouse serum inhibits the detection of IVIg-specific mouse IgG by ELISA but not the detection of total mouse IgG. Blocking buffer, IVIg or bevacizumab were added at 10, 20 or 50 mg/mL in serum from mice treated 3 times weekly with 1mg of IVIg (n = 3). Statistical significance was tested using two-way ANOVA (Dunnett’s test) where each group was compared to the control group ‘Buffer’. (F) ELISA measuring mouse IgG anti-IVIg-Fc and (Fab)’_2_ fragments. Data pool 2 independent experiments (n = 6). Bars represent mean ± SD. Star maker significant difference *: p < 0.05; **p<0.01; ***: p<0.001; ****: p<0.0001. n.d: not detected.

After demonstrating that IVIg reduced the Ova-specific antibody titer, the ability of IVIg to impair the frequency of immunoglobulin-secreting cells (ISC) *in vivo* was assessed by ELISPOT. As expected, the proportion of Ova-specific antibody-secreting cells (ASC) relative to the total IgG ISC (Fig 5A) seemed to be reduced upon IVIg administration, but due to the high inter-animal variability, the effect was not statistically significant. IVIg significantly increased the number of total IgG ISC the draining lymph nodes, compared to Ova-immunized mice (Fig 5B), while total IgM-ISC (Fig 5C) were not affected. These data might possibly reflect a massive recruitment of IVIg-specific B cells and their further differentiation into IgG-secreting plasma cells and a less efficient mobilization of Ova-specific immune cells.

Different doses of IVIg ranging from 1 mg up to 50 mg per animal were tested to further explore the effect on the formation of IVIg-specific IgG and total mouse IgG. Injection of 1 mg of IVIg led to a prominent IVIg-specific immune response in mice, and increased the total mouse IgG titer compared to the Ova-immunized group (Fig 5D). Surprisingly, diminishing levels of both IVIg-specific- and total- mouse IgG were detected in mouse serum when the dose of IVIg administered was increased. To determine if there was an interference in the assay, a competition assay was performed by spiking different concentrations of either IVIg or bevacizumab into the serum of mice that were treated with 1 mg IVIg (Fig 5E). There was no inhibition in the detection signal of total mouse IgG following IVIg or bevacizumab spiking compared to buffer spiking. This suggests that IVIg accelerated the clearance of mouse IgG most likely via FcRn saturation. However, addition of IVIg strongly inhibited the detection of specific anti-IVIg mouse IgG for all doses tested. This indicates that the reduction in signal upon increasing doses of IVIg may be explained by assay interference, as higher doses of administered IVIg enhanced complexing with anti-IVIg mouse antibodies in the serum, thereby inhibiting their detection via ELISA. In contrast, spiking of bevacizumab led to a moderate signal inhibition and only at the maximal dose (Fig 5E). Taken together these data suggest that mice develop antibodies targeting both Fc and Fab regions of IVIg.

To further confirm the specificity of anti-IVIg mouse IgG, ELISA was performed after coating purified IVIg-Fc or IVIg-(Fab’)_2_ fragments. Titers of mouse IgG recognizing the (Fab’)_2_ fragments from IVIg were decreased upon increasing doses of IVIg administered (Fig 5F), indicating assay interference. In contrast, levels of mouse anti-Fc antibodies did not show significant changes upon different doses of administered IVIg, suggesting only weak assay interference and thereby low concentrations of mouse anti-Fc antibodies in the sera. Overall these results reveal that IVIg raised a specific antibody response in mice mainly directed towards the Fab region, which contains the highest degree of sequence diversity.

### IVIg does not decrease the antibody response against a thymus-independent antigen but recruits mature B cells to draining lymph nodes

**Fig 6:**
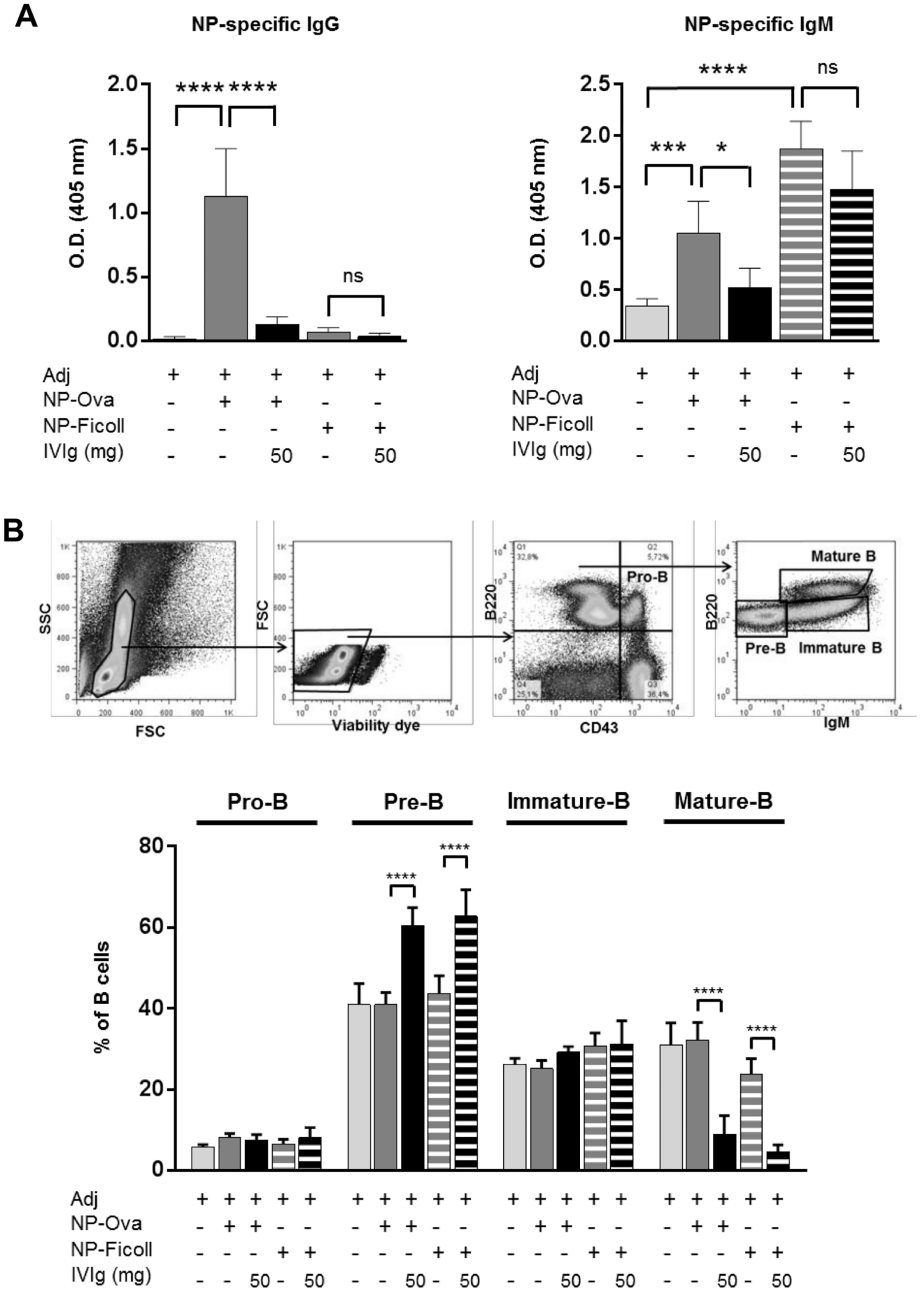
IVIg does not decrease the antibody response against a thymus-independent antigen but recruits mature B cells to draining lymph nodes. Mice were immunized with NP-OVA or NP-Ficoll together with adjuvant AddaVax^®^, with or without IVIg as indicated. (A) ELISA for NP-specific mouse IgG (serum diluted 1:500) and for NP-specific mouse IgM (serum diluted 1:50). Data are derived from one experiment (n = 6). Statistical significance was tested using one-way ANOVA (Tukey’s test) where each group was compared with every other. (B) IVIg does not affect the B-cell compartment in the spleen but reduces the proportion of mature re-circulating B cells in the bone-marrow and promotes pre-B cell formation. Gating strategy is shown for the different B-cell compartments in the bone-marrow. Statistical significance was tested using two-way ANOVA (Tukey’s test) where each group was compared with every other. Data are from one experiment (n = 6). Bars represent mean ± SD. Star maker significant difference *: p < 0.05; **p<0.01; ***: p<0.001; ****: p<0.0001. ns: not significant.

To address whether the effect of IVIg was thymus (i.e. T cells) dependent, NP-Ficoll or NP-Ova were co-administered to IVIg. In mice immunized with the thymus dependent antigen NP-Ova, IVIg administration efficiently decreased the production of NP-specific IgG by 88% and NP-specific IgM by 51% (Fig 6A). In mice immunized with the thymus-independent antigen, NP-Ficoll, the NP-antibody response was mainly of IgM isotype, as expected, and IVIg injections did not significantly reduce the NP-specific IgM titer. These results demonstrate that IVIg has minimal if any effect on the antibody response towards a thymus-independent antigen and suggests that involvement of T cells is essential for IVIg to exert its inhibitory effect on the production of antigen-specific antibodies.

To investigate the effect of IVIg on different B cell populations, the distribution of the different B-cell compartments in the bone marrow was investigated (Fig 6B). Administration of IVIg together with adjuvant significantly increased the proportion of pre-B cells in the bone-marrow and strongly reduced the percentage of re-circulating mature B cells. This indicates that IVIg recruits a large number of mature B cells to the draining lymph nodes, thus preventing their re-circulation to the bone-marrow. This is in agreement with the increased number of B cells in peripheral lymphoid tissues upon IVIg treatment previously observed (Fig 1D).

## Discussion

Here we demonstrate that high doses of IVIg co-injected with Ova and adjuvant induce major changes in the response towards Ova and also affect the distribution and activation state of multiple lymphocytes populations. The observed changes encompass i) an IVIg-dose-dependent upregulation of the co-stimulatory molecules CD86 and CD83 on B cells, as well as the early activation marker CD69 on both B cells and CD4+ T cells; ii) a strong reduction in the number of re-circulating mature B cells in the bone marrow indicating recruitment of these cells to the lymphoid organs; iii) a marked increase in weight of the peripheral lymphoid organs, associated with increased numbers of both B and CD4+ T cells; iv) the formation of large and numerous germinal centers in the draining lymph nodes and in the spleen upon IVIg administration; v) production of specific IgG subclass anti-IVIg antibodies, specific for IVIg-Fab regions. Together, our data suggest a massive lymphocyte activation by IVIg in mice.

Common side effects that occur in patients affected with autoimmune disorders and treated with high dose IVIg therapy include fever, chills, rash, fatigue, flushing, muscle pain [50–52] or even swelling of cervical lymph nodes [53]. Strikingly, these are typical symptoms of an inflammation and are possibly reflecting a strong anti-IVIg immune response in patients. For all of the effects observed in mice, the co-injection of adjuvant with IVIg was required, which is in line with the fact that an activation signal of the innate immune system is required to efficiently prime an adoptive immune response. Still, IVIg is administered in patients without adjuvant. However, the chronic inflammation in autoimmune diseases could provide “adjuvant-like” activation signals, which could be sufficient to activate B and T cells specific for IVIg. Still, caution must be exercised in the translatability of our findings to human as mouse models are not always appropriate to study mechanisms for phenomenon occurring in humans.

Our data are in line with studies showing that IVIg stimulates B cells [54] and CD4+ T cells *in vivo* and *in vitro* [54, 55], and increased *in vitro* secretion of IgG [56, 57] that were highly reactive against human IgG F(ab')_2_ fragments [57]. Importantly, Fab fragments isolated from one Kawasaki patient before and after IVIg treatment revealed that they displayed a higher affinity for IVIg after therapy [58].

Data from marketed human monoclonal IgGs bevacizumab and panitumumab used in IVIg equivalent doses as “IVIg surrogates” provide important insights into the mechanisms of IVIg: i) IVIg decreased the level of total mouse IgG measured in serum in a dose-dependent manner, suggesting that IVIg increased antibody clearance via FcRn blocking as postulated by other groups [15]. However, both monoclonal antibodies failed to decrease the titer of Ova-specific antibodies in mice, suggesting that FcRn saturation doesn’t seem to be the main driver of the inhibitory effects of IVIg in the Ova-specific response. Moreover, neither bevacizumab nor panitumumab triggered activation of B and T cells in Ova-immunized mice as it was observed with IVIg. ii) IVIg and the two monoclonal antibodies contain identical constant regions including the sequences proposed to act as regulatory T cell epitopes [59], but only IVIg induced the downregulation of the anti-OVA response. This argues against the idea that regulatory T cell epitopes are the driver of the observed effects. This is in line with our recent study showing that regulatory T cell epitopes are not efficiently presented by antigen presenting cells [60].

So why is IVIg so different from a monoclonal antibody? Apart from differences in glycosylation, the biggest difference between IVIg and monoclonal antibodies is the enormous diversity of sequences from the variable regions in the IVIg preparations, which are derived from several thousand donors. Due to the differences in human and mouse IgG sequences, we cannot exclude that the mouse response against human constant domains of IVIg is involved, but our data strongly suggest that the variable domains of IVIg may still have a significant contribution in the massive immune response observed *in vivo* in mice.

**Fig 7:**
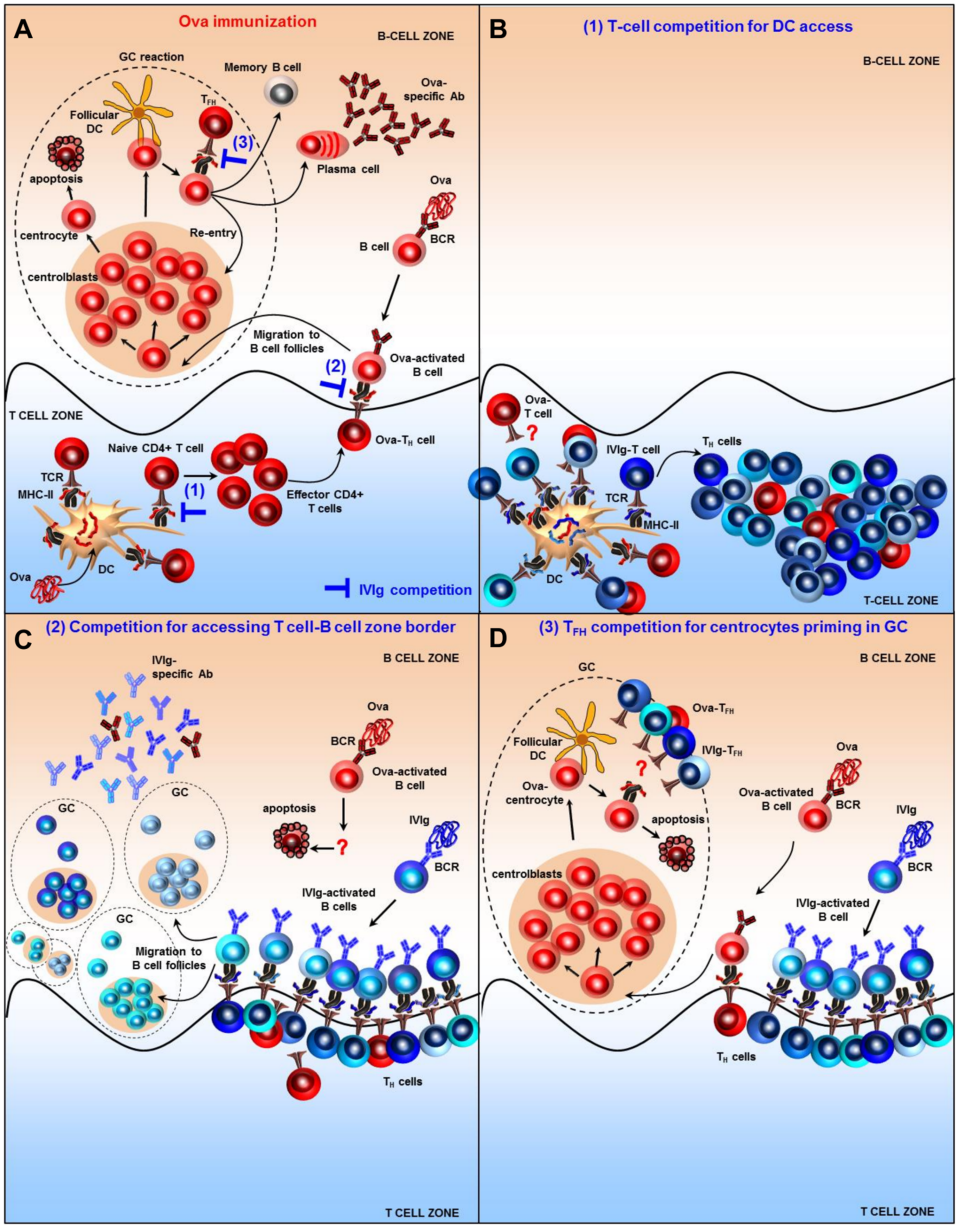
A proposed mechanism of action of IVIg based on T-cell competition effects occurring at several stages of the adaptive immune response. (A) Classical immune response and germinal center formation after Ova immunization. T cells primed by Ova-derived epitopes presented on APCs differentiate into effector T helper cells (T_H_) and migrate at the T cell–B cell border of the lymph node. Ova-activated B cells take up and present antigenic peptides to T_H_. After receiving co-stimulatory signals they initiate the formation of a germinal center (GC). After a cycle of proliferation and somatic hypermutations in the dark zone, B cells moves to the light zone to meet with their cognate antigen exposed on follicular dendritic cells (FDCs). If the affinity of the mutated B cell receptor (BCR) is very low, the B cell will not receive survival signals and will undergo apoptosis. The surviving B cells need to compete for help from T follicular helper cells (T_FH_), thus favoring B cells with high affinity BCRs. B cells can then either re-enter the dark zone to further mature the BCR affinity, or exit the GC as plasma cells or memory B cells. Blue lines depict IVIg potential competition mechanisms. (B) T-cell competition for DCs access. In addition to Ova-peptides, DCs present a multitude of diverse IVIg-derived epitopes. This favors the competition between the many different IVIg-specific T cell clones with the Ova-T cell clones to receive priming from DCs. (C) Competition for accessing T cell-B cell zone border. The few Ova-T cell clones that may have been primed by DCs will be outnumbered by the numerous IVIg-T_H_ clones accessing the T cell-B cell zone border. This reduces the likelihood of OVA-T cell clones engaging with their cognate Ova-B cells. Likewise IVIg-B cells will spatially compete with Ova-specific B cells for accessing their cognate T_H_ cells. This results in apoptosis of Ova-activated B cells. (D) T_FH_ competition for centrocytes priming in GC. Only few Ova-T cell clones will be able to be primed by DCs, deliver helper signals to their cognate Ova-activated B cells and induce the formation of a GC. However, the help delivered by T_FH_ to centrocytes in GC is very limited. As the numerous IVIg- T_FH_ will compete with the few Ova- T_FH_ for access to centrocytes, the latter will be more likely to die by apoptosis.

Based on our findings, we propose a new model that may explain the immunomodulatory properties of IVIg and that is based on antigenic competition at the T-cell level rather than induction of regulatory T cells. Fig 7A illustrates the classical course of the Ova-adaptive immune response, with blue symbols indicating where IVIg may potentially interfere. Competition between T-cells can arise for access to antigen presenting cells (APCs) (Fig 7B). Several studies have shown that a competition between T cells occurs if target peptides are presented on the same APC, most likely via steric hindrance [61–64]. This is amplified by the fact that the contact time of T cells recognizing their cognate peptides on APCs is longer than for unrelated peptides [65]. IVIg induces the presentation of highly diverse IVIg-peptides via MHC class II molecules [60] and T cell clones specific for IVIg may out-compete Ova-specific T cells regarding spatial access to the DCs. In contrast, competition of IVIg peptides and OVA-peptides for MHC class II binding and presentation is unlikely, since it has been demonstrated that T-cell competition does not result from diminished presentation of peptides [62, 64], and that even high doses of IVIg do not impair the presentation of antigenic peptides on MHC class II [60]. T-cell competition may also occur at the T cell-B cell zone border (Fig 7C). There, numerous IVIg-specific T-helper clones interacting with their cognate B cells may prevent efficient T-cell priming of Ova-activated B cells. Similarly, competition between T-follicular helper (T_FH_) for priming of centrocytes in the germinal center may occur (Fig 7D), as T_FH_ have been demonstrated to be present in a limiting amount to ensure competition amongst the different B cells clones and optimal affinity maturation [66].

Antigenic competition has been described in several studies and was found to be enhanced when larger doses of antigen were used [67–69]. This supports the model presented here and may explain the requirement of high doses of IVIg to treat autoimmune diseases. This model is further reinforced by studies showing that IVIg treatment decreased Ova-specific T-cell activation and proliferation, both *in vitro* [70] and *in vivo* [71].

Although research over the past decade has emphasized the role of IVIg in promoting anti-inflammatory effects, our data reveal that IVIg induces a massive immune reaction in mice that interferes with establishing efficient immune responses against other antigens.

We propose that IVIg comprises the ability to re-direct the immune system to react against the multitude of epitopes it contains. Whether this finding obtained *in vivo* in mice can be correlated to the human situation remains to be further investigated in IVIg-treated patients’ samples. Owing to the complexity of IVIg preparations it is likely that multifactorial and non-mutually exclusive mechanisms are involved in its immunomodulatory effect.

## Supporting information

S1 Fig IVIg has negligible effect on CD80, CD40 and I-A expression on B cells. Mice were immunized with Ova as described previously and 50 mg of IVIg were injected simultaneously. Data pool 4 independent experiments (n = 12) for CD40 and CD80 or 6 independent experiments (n = 18) for I-A. Statistical significance was tested using one-way ANOVA (Dunnett’s test). Bars represent mean ± SD. *: p < 0.05; **p<0.01; ***: p<0.001; ****: p<0.0001. LN: lymph nodes. MFI: Median of fluorescence intensity.
(PDF)

S2 Fig IVIg has no effect on DCs and Tregs phenotype. Mice were immunized with Ova as described previously and 50 mg of IVIg were co-injected. (A) DCs were defined as CD11c+ I-A+ cells. Flow cytometry histograms of co-stimulatory molecules expressed on DCs in the spleen are shown for one representative animal. (B) Tregs were defined as CD3+ CD4+ CD25+ Foxp3+ cells. Percentage of Tregs was assessed in the spleen and draining lymph nodes. The table indicates MFI (median of fluorescence intensity) values for Treg activation markers in the IVIg-treated group. Values are expressed as ratio to the ‘Adj + Ova’ treated group ± SD. Data pool 4 independent experiments (n = 12). Statistical significance was tested using one-way ANOVA (Dunnett’s test). LN: lymph nodes.
(PDF)

S3 Fig IVIg effects are antigen-independent and could not be reproduced by IVIg formulation buffer. (A) Mice were immunized with 50 μg BSA as described previously for Ova and 50 mg of IVIg were co-injected. BSA-specific IgG antibodies were measured by ELISA and expression of CD86 and CD83 on B cells was assessed by flow cytometry. Data are from one experiment (n = 3). Statistical significance was tested using one-way ANOVA (Dunnett’s test). (B) Mice were immunized with Ova and 50 mg of IVIg or the equivalent volume of IVIg formulation buffer (obtained by filtering IVIg through a 30-kDa filter) were co-injected. Results show anti-Ova IgG measured by ELISA, weight measurements, and flow cytometry measurements performed on lymphoid organs. Data are derived from one experiment (n = 3). Statistical significance was tested using one-way ANOVA (Dunnett’s test). Bars represent mean ± SD. *: p < 0.05; **p<0.01; ***: p<0.001; ****: p<0.0001. n.d: not detected. ns: not significant. MFI: Median of fluorescence intensity. LN: lymph nodes.
(PDF)

S4 Fig IVIg inhibits the formation of Ova-specific antibodies and activates B cells irrespectively of the type of adjuvant co-administered. Mice were immunized with Ova and 50 mg of IVIg were co-injected. Five different adjuvants were tested as indicated. (A) Ova-specific mouse IgG_1_ were measured by ELISA and expressed as relative units to standard or as ratio to ‘Adj + Ova’ group. n = 12 for Adjuvant LT(R192G/L211A) (3 independent experiments); n = 3 for CpG; n = 3 for MPLA-SM; n = 6 for aluminum (2 independent experiments); n = 9 for AddaVax^®^ (3 independent experiments). (B) Results from weight measurement and flow cytometry analyses. n = 12 for Adjuvant LT(R192G/L211A) (3 independent experiments); n = 3 for CpG; n = 3 for MPLA-SM; n = 6 for aluminum (2 independent experiments) for spleen and axillary/brachial LN; n = 3 for aluminum for cervical LN; n = 12 for AddaVax^®^ (4 independent experiments). Bars represent mean ± SD. Statistical significance was tested using two-way ANOVA (Dunnett’s test). *: p < 0.05; **p<0.01; ***: p<0.001; ****: p<0.0001. ns: not significant. MFI: Median of fluorescence intensity. LN: lymph nodes.
(PDF)

S5 Fig Panitumumab, a humanized monoclonal IgG_2_κ antibody, does not reproduce IVIg effects. Mice were immunized with Ova as described previously using either adjuvant AddaVax^®^ (MF59) or adjuvant LT(R192G/L211A). IVIg or panitumumab were co-injected as indicated. (A) Anti-Ova IgGs in mouse serum were measured by ELISA. Values are expressed as relative units to standard. Results are derived from one experiment (n = 3). Statistical significance was tested using one-way ANOVA (Dunnett’s test). (B) Draining lymph nodes and spleen were harvested, weighed and flow cytometry was performed on isolated cells (adjuvant LT(R192G/L211A) was used). Data are derived from one experiment (n = 3). Bars represent mean ± SD. Statistical significance was tested using one-way ANOVA (Dunnett’s test). *: p< 0.05; **p<0.01; ***: p<0.001; ****: p<0.0001. ns: not significant. MFI: Median of fluorescence intensity. LN: lymph nodes. n.d: not detected.
(PDF)

S6 Fig Count of germinal centers. Spleen and draining lymph nodes were stained for germinal centers with GL7 antibody (green). Each germinal center is delimited by a red circle. Each picture is generated from one representative animal. n = 6 animals from 2 independent experiments for (B) and n = 3 animals for (C). Magnification: x 0.25.
(PDF)
