# Supplementary figures and images for: Massive immune response against IVIg interferes with response against other antigens in mice: A new mode of action?
Source: PLoS One. 2017 Oct 12;12(10):e0186046. doi: 10.1371/journal.pone.0186046 (PMC5638328; doi:10.1371/journal.pone.0186046)

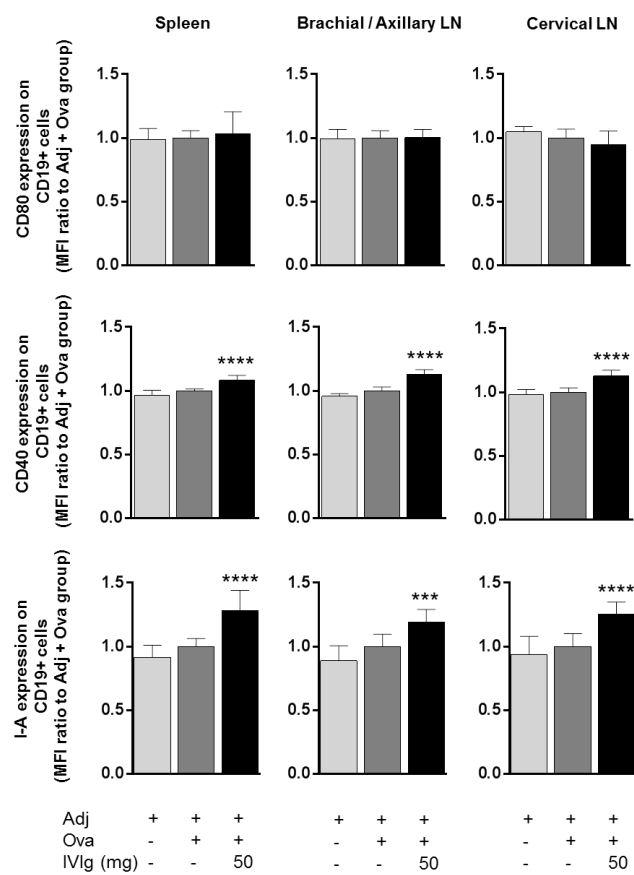

Supplement: S1 Fig — Mice were immunized with Ova as described previously and 50 mg of IVIg were injected simultaneously. Data pool 4 independent experiments (n = 12) for CD40 and CD80 or 6 independent experiments (n = 18) for I-A. Statistical significance was tested using one-way ANOVA (Dunnett’s test). Bars represent mean ± SD. *: p < 0.05; **p<0.01; ***: p<0.001; ****: p<0.0001. LN: lymph nodes. MFI: Median of fluorescence intensity. (PDF) [file pone.0186046.s001.pdf]

A

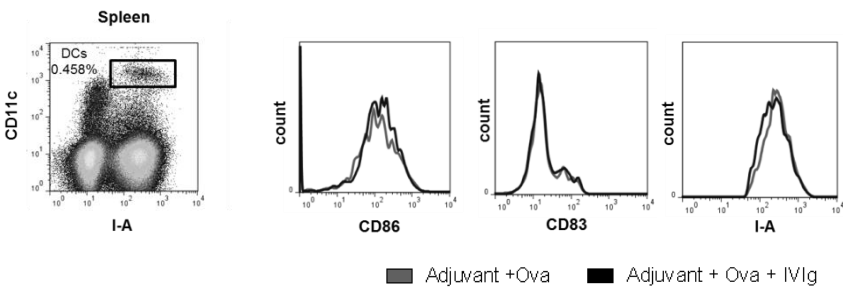

B

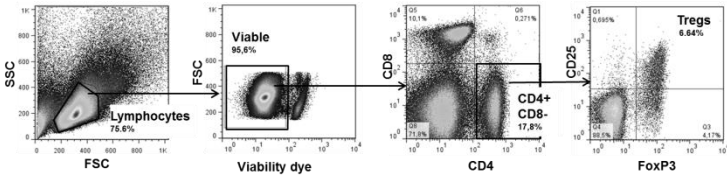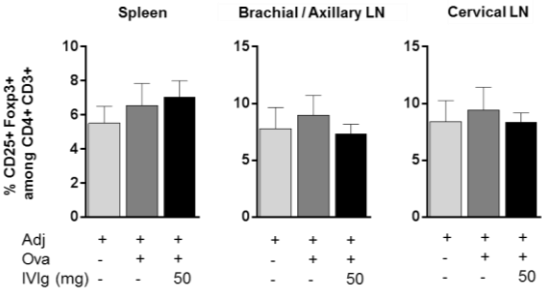

|                       | Foxp3       | CTLA-4      | CD25        |
|-----------------------|-------------|-------------|-------------|
| spleen                | 1.11 ± 0.05 | 1.15 ± 0.12 | 1.22 ± 0.16 |
| axillary/ brachial LN | 1.03 ± 0.03 | 1.11 ± 0.13 | 1.17 ± 0.15 |
| cervical LN           | 1.03 ± 0.05 | 1.02 ± 0.18 | 1.11 ± 0.23 |

Supplement: S2 Fig — Mice were immunized with Ova as described previously and 50 mg of IVIg were co-injected. (A) DCs were defined as CD11c+ I-A+ cells. Flow cytometry histograms of co-stimulatory molecules expressed on DCs in the spleen are shown for one representative animal. (B) Tregs were defined as CD3+ CD4+ CD25+ Foxp3+ cells. Percentage of Tregs was assessed in the spleen and draining lymph nodes. The table indicates MFI (median of fluorescence intensity) values for Treg activation markers in the IVIg-treated group. Values are expressed as ratio to the ‘Adj + Ova’ treated group ± SD. Data pool 4 independent experiments (n = 12). Statistical significance was tested using one-way ANOVA (Dunnett’s test). LN: lymph nodes. (PDF) [file pone.0186046.s002.pdf]

**A**

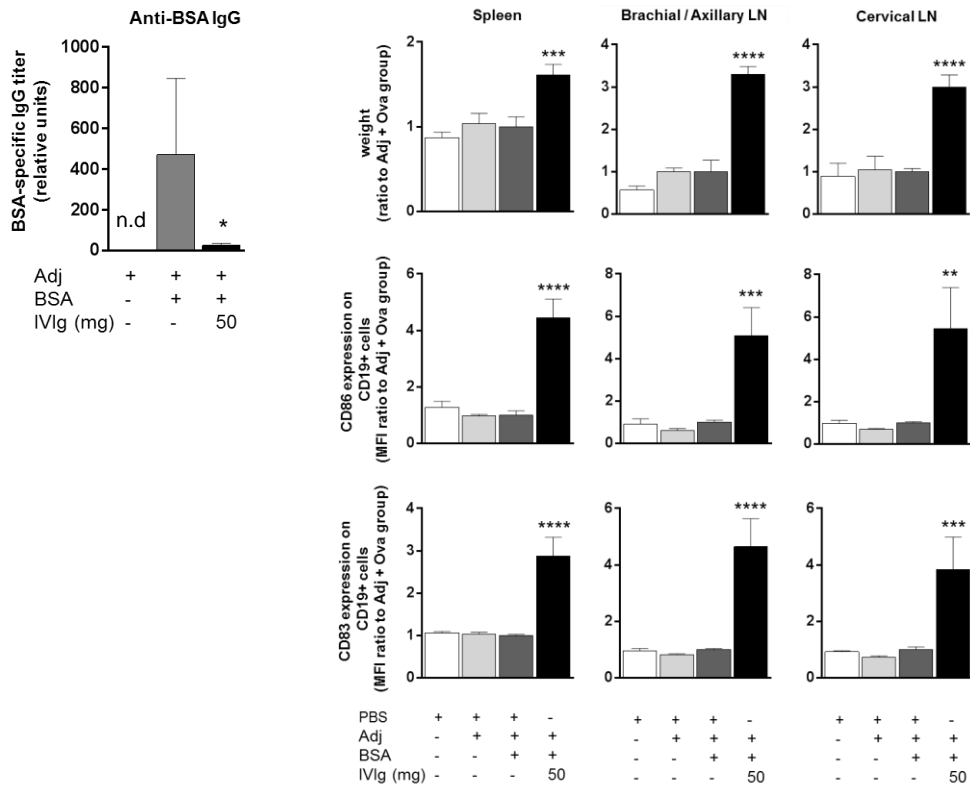

**B**

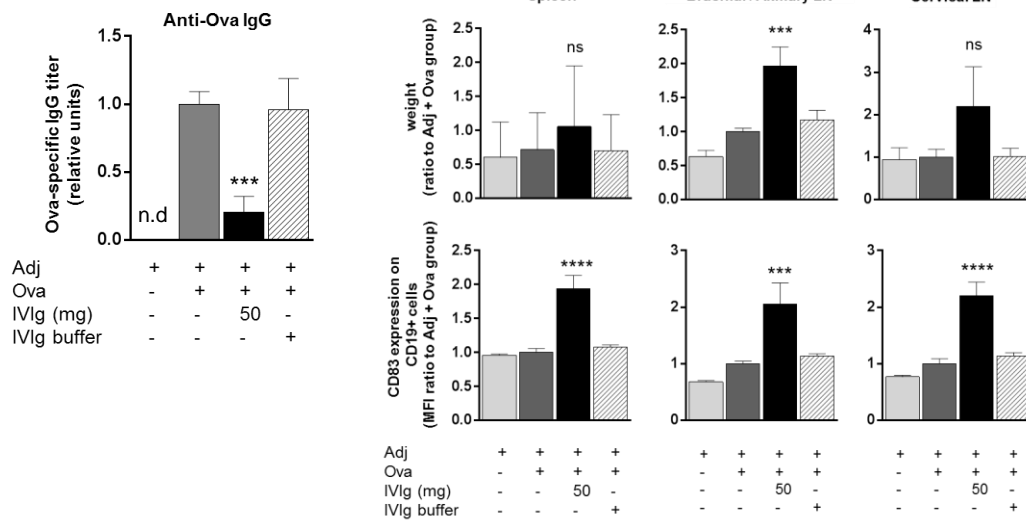

Supplement: S3 Fig — (A) Mice were immunized with 50 μg BSA as described previously for Ova and 50 mg of IVIg were co-injected. BSA-specific IgG antibodies were measured by ELISA and expression of CD86 and CD83 on B cells was assessed by flow cytometry. Data are from one experiment (n = 3). Statistical significance was tested using one-way ANOVA (Dunnett’s test). (B) Mice were immunized with Ova and 50 mg of IVIg or the equivalent volume of IVIg formulation buffer (obtained by filtering IVIg through a 30-kDa filter) were co-injected. Results show anti-Ova IgG measured by ELISA, weight measurements, and flow cytometry measurements performed on lymphoid organs. Data are derived from one experiment (n = 3). Statistical significance was tested using one-way ANOVA (Dunnett’s test). Bars represent mean ± SD. *: p < 0.05; **p<0.01; ***: p<0.001; ****: p<0.0001. n.d: not detected. ns: not significant. MFI: Median of fluorescence intensity. LN: lymph nodes. (PDF) [file pone.0186046.s003.pdf]

**A**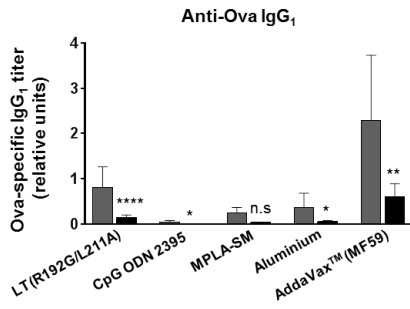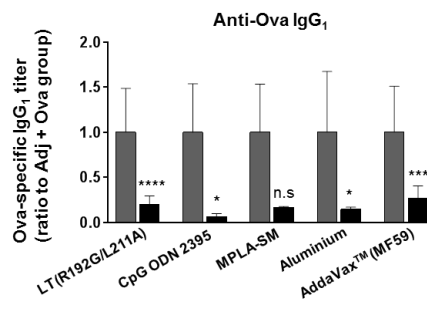**B**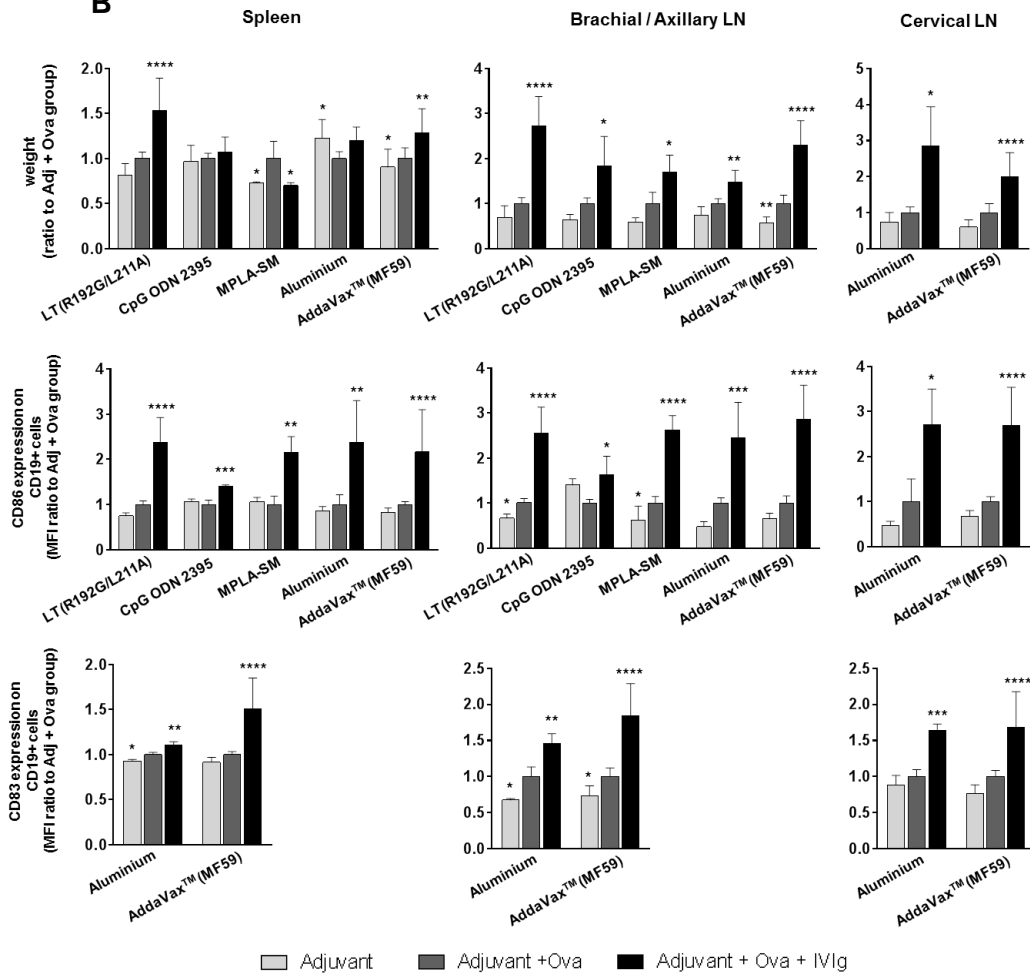

Supplement: S4 Fig — Mice were immunized with Ova and 50 mg of IVIg were co-injected. Five different adjuvants were tested as indicated. (A) Ova-specific mouse IgG1 were measured by ELISA and expressed as relative units to standard or as ratio to ‘Adj + Ova’ group. n = 12 for Adjuvant LT(R192G/L211A) (3 independent experiments); n = 3 for CpG; n = 3 for MPLA-SM; n = 6 for aluminum (2 independent experiments); n = 9 for AddaVax® (3 independent experiments). (B) Results from weight measurement and flow cytometry analyses. n = 12 for Adjuvant LT(R192G/L211A) (3 independent experiments); n = 3 for CpG; n = 3 for MPLA-SM; n = 6 for aluminum (2 independent experiments) for spleen and axillary/brachial LN; n = 3 for aluminum for cervical LN; n = 12 for AddaVax® (4 independent experiments). Bars represent mean ± SD. Statistical significance was tested using two-way ANOVA (Dunnett’s test). *: p < 0.05; **p<0.01; ***: p<0.001; ****: p<0.0001. ns: not significant. MFI: Median of fluorescence intensity. LN: lymph nodes. (PDF) [file pone.0186046.s004.pdf]

**A**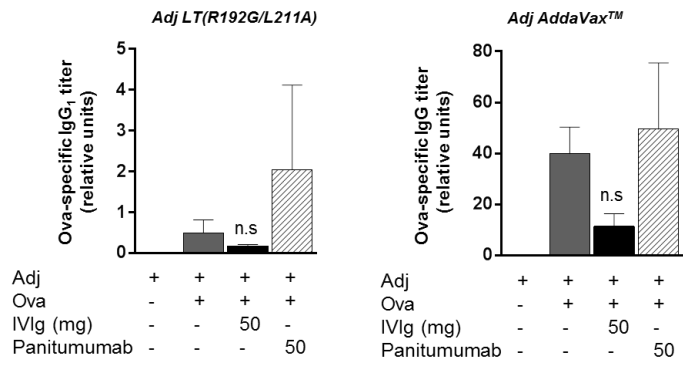**B**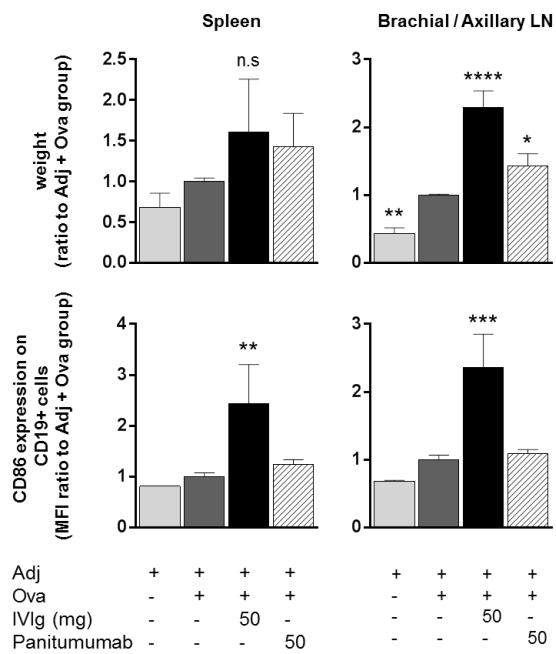

Supplement: S5 Fig — Mice were immunized with Ova as described previously using either adjuvant AddaVax® (MF59) or adjuvant LT(R192G/L211A). IVIg or panitumumab were co-injected as indicated. (A) Anti-Ova IgGs in mouse serum were measured by ELISA. Values are expressed as relative units to standard. Results are derived from one experiment (n = 3). Statistical significance was tested using one-way ANOVA (Dunnett’s test). (B) Draining lymph nodes and spleen were harvested, weighed and flow cytometry was performed on isolated cells (adjuvant LT(R192G/L211A) was used). Data are derived from one experiment (n = 3). Bars represent mean ± SD. Statistical significance was tested using one-way ANOVA (Dunnett’s test). *: p< 0.05; **p<0.01; ***: p<0.001; ****: p<0.0001. ns: not significant. MFI: Median of fluorescence intensity. LN: lymph nodes. n.d: not detected. (PDF) [file pone.0186046.s005.pdf]

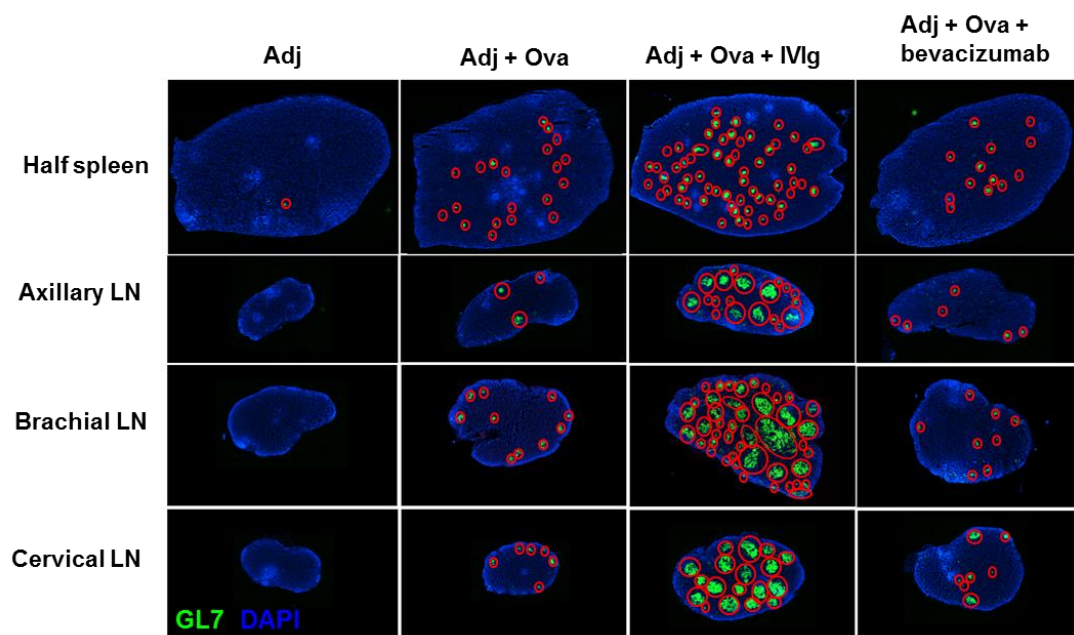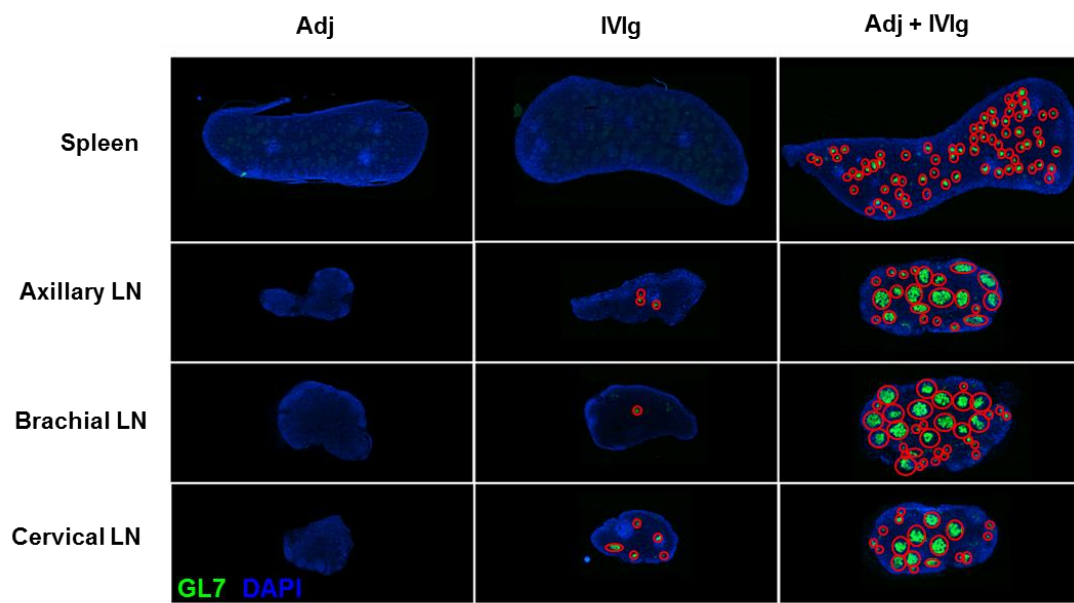

Supplement: S6 Fig — Spleen and draining lymph nodes were stained for germinal centers with GL7 antibody (green). Each germinal center is delimited by a red circle. Each picture is generated from one representative animal. n = 6 animals from 2 independent experiments for (B) and n = 3 animals for (C). Magnification: x 0.25. (PDF) [file pone.0186046.s006.pdf]
